# Differential response of cassava genotypes to infection by cassava mosaic geminiviruses

**DOI:** 10.1016/j.virusres.2016.09.022

**Published:** 2017-01-02

**Authors:** Paul Kuria, Muhammad Ilyas, Elijah Ateka, Douglas Miano, Justus Onguso, James C. Carrington, Nigel J. Taylor

**Affiliations:** a Jomo Kenyatta University of Agriculture and Technology, PO Box 62000-00200 Nairobi, Kenya; b Kenya Agricultural and Livestock Research Organization, PO Box 57811-00200, Nairobi, Kenya; c Donald Danforth Plant Science Center, 975 North Warson Road, St. Louis, MO, 63132, USA; d University of Nairobi, PO BOX 30197, 00100, Nairobi, Kenya

**Keywords:** African cassava mosaic virus, East african cassava mosaic virus, Cassava mosaic disease, CMD1, CMD2, CMD3

## Abstract

Mitigation of cassava mosaic disease (CMD) focuses on the introgression of resistance imparted by the polygenic recessive (CMD1), dominant monogenic (CMD2) and CMD3 loci. The mechanism(s) of resistance they impart, however, remain unknown. Two CMD susceptible and nine CMD resistant cassava genotypes were inoculated by microparticle bombardment with infectious clones of *African cassava mosaic virus* Cameroon strain (ACMV-CM) and the Kenyan strain K201 of *East African cassava mosaic virus* (EACMV KE2 [K201]). Genotypes carrying the CMD1 (TMS 30572), CMD2 (TME 3, TME 204 and Oko-iyawo) and CMD3 (TMS 97/0505) resistance mechanisms showed high levels of resistance to ACMV-CM, with viral DNA undetectable by PCR beyond 7 days post inoculation (dpi). In contrast, all genotypes initially developed severe CMD symptoms and accumulated high virus titers after inoculation with EACMV KE2 (K201). Resistant genotypes recovered to become asymptomatic by 65 dpi with no detectable virus in newly formed leaves. Genotype TMS 97/2205 showed highest resistance to EACMV KE2 (K201) with <30% of inoculated plants developing symptoms followed by complete recovery by 35 dpi. Deep sequencing of small RNAs confirmed production of 21–24 nt virus derived small RNAs (vsRNA) that mapped to cover the entire ACMV-CM and EACMV KE2 (K201) viral genomes in both polarities, with hotspots seen within gene coding regions. In resistant genotypes, total vsRNAs were most abundant at 20 and 35 dpi but reduced significantly upon recovery from CMD. In contrast, CMD susceptible genotypes displayed abundant vsRNAs throughout the experimental period. The percentage of vsRNAs reads ranked by class size were 21nt (45%), 22 nt (28%) and 24 nt (18%) in all genotypes studied. The number of vsRNA reads directly correlated with virus titer and CMD symptoms.

## Highlights

• Cassava genotypes respond differently to infection by cassava mosaic geminiviruses.
• Cassava mosaic disease resistant loci prompt recovery from systemic infection.
• CMD symptoms are directly correlated with contents of viral DNA and virus specific small RNAs.
• CMD infected plants abundantly accumulate 21–24 nt of virus specific small RNAs.
• VsRNAs heterogeneously map the entire virus genome in both polarities.

## 1 Introduction

Cassava (*Manihot esculenta* Crantz) is a tropical woody perennial shrub of the family Euphorbiaceae, grown for its starchy storage roots. Under optimum conditions, maximum yield potential is 50 Mt (MT) of fresh storage roots per hectare (FAOSTAT, 2014). However, abiotic and biotic stress, including pests and diseases, limit production to an average of 15 tons per hectare worldwide and only 10.9 in Africa (FAOSTAT, 2014, Legg et al., 2015). In sub-Saharan Africa, cassava production is constrained by cassava mosaic disease (CMD) and cassava brown streak disease (CBSD) (Legg et al., 2015, Patil and Fauquet, 2015a, Patil et al., 2015b). CMD occurs as a complex of different whitefly-transmitted cassava mosaic geminiviruses (CMGs) (genus begomovirus, family Geminiviridae) (Patil and Fauquet, 2009). CMD is prevalent in all cassava growing regions of sub-Saharan Africa and the Indian sub-continent (Legg et al., 2011, Patil and Fauquet, 2009), with eleven species characterized worldwide (Legg et al., 2015, Patil and Fauquet, 2009).

CMGs are composed of two circular single stranded DNA (ssDNA) genomic components, namely DNA A and DNA B, ranging in size from 2.7–3.0 kb (Briddon et al., 2010, Hanley-Bowdoin et al., 2013). DNA A encodes two overlapping virion-sense open reading frames (ORFs) involved in encapsidation (AV1) and silencing suppressor targeting post-transcriptional gene silencing (PTGS) (AV2 or V2) (Glick et al., 2008, Zhang et al., 2012), four partially overlapping complementary-sense OFRs necessary for replication (AC1, AC3) and for transcription and suppression of host mediated gene silencing (AC2, AC4) (Jeske, 2009). DNA B encodes two non-overlapping ORFs (BC1, BV1), each present on the complementary and virion strands, that are involved in inter- and intracellular trafficking of the virus (Hanley-Bowdoin et al., 2013). The rightward and leftward transcriptional units of the DNA A and DNA B components, respectively, are separated by a homologous intergenic region (IR) of approximately 200 nucleotides (Bull et al., 2007, Hanley-Bowdoin et al., 2013).

Breeding has delivered high yielding cassava varieties that are resistant or tolerant to CMGs (Okogbenin et al., 2012, Rabbi et al., 2014). Presently, three genetically distinct CMD resistance/tolerance mechanisms have been described in cassava (Okogbenin et al., 2012, Rabbi et al., 2014). The CMD1 type resistance mechanism was introgressed from *Manihot glaziovii* Muell. Arg. (ceara rubber) and has been reported to be polygenic and recessive in nature (Fregene and Puonti-Kaerlas, 2002). In contrast, CMD2 type resistance is derived from a single genetic locus and is found in different accessions of West African landraces of the tropical *Manihot esculenta* (TME) series (Akano et al., 2002, Rabbi et al., 2014). Breeding programs in Africa and Latin America have preferentially exploited the CMD2 locus to develop highly CMD resistant genotypes, as it is easily heritable and consistently imparts stable resistance to a broad spectrum of CMGs (Okogbenin et al., 2013, Rabbi et al., 2014). Recently, a new CMD resistant, designated CMD3, was described in the elite cultivar TMS 97/2205 (Okogbenin et al., 2012). TMS 97/2205 was derived from crosses of TMS 30572 (CMD1 resistant type) and TME 6 (CMD2 resistant type) (Dixon et al., 2010). Field reports indicate that TMS 97/2205 is highly resistant to CMD with less than 1% disease incidence occurring under high disease pressure in Nigeria (Okogbenin et al., 2012). Genetic studies conducted on TMS 97/2205 reveal the presence of the CMD2 locus and an additional locus in the same linkage group (Okogbenin et al., 2012).

Under field conditions, genotypes containing CMD1, CMD2 and CMD3 loci develop moderate to severe CMD symptoms followed by complete recovery from disease (Okogbenin et al., 2012). Importantly, however, the genes and the underlying molecular mechanisms conferring recovery from CMD, and the resistance imparted by CMD1, CMD2 and CMD3, have not been elucidated.

Antiviral defense mechanisms associated with RNA silencing have been reported in plants seen to display the recovery phenotype, leading to clearance of the viral RNA from infected plants (Butterbach et al., 2014, Jovel et al., 2007, Ma et al., 2015). Processing of double stranded RNA (dsRNA) into small RNA (sRNA) duplexes of 21–24 nucleotides is governed by RNase III-like Dicer-like (DCL) enzymes (Bologna and Voinnet, 2014). The sRNAs are incorporated into the Argonaute (AGO) protein complex to direct slicing and degradation of RNA molecules with sequence homology to the AGO-bound sRNAs (Carbonell and Carrington, 2015, Kobayashi and Tomari, 2016, Meister, 2013). The *Arabidopsi*s *thaliana* genome encodes four DCL and ten AGO families, many of which participate in antiviral defense (Carbonell and Carrington, 2015, Garcia-Ruiz et al., 2015). Activity of DCL4, DCL2 and DCL3 leads to biogenesis of virus specific sRNAs of 21, 22 and 24 nt sizes, respectively, that act as primary defense against viruses (Aregger et al., 2012, Blevins et al., 2006, Parent et al., 2015). Viruses, in turn, encode suppressors of gene silencing (VSRs) that interact with major elements of the plant silencing machinery and subvert antiviral defense (Burgyan and Havelda, 2011). The role of VSRs in symptom recovery has been demonstrated and linked to RNA silencing, whereby plants infected with suppressor deficient viruses induce resistance resembling recovery (Carbonell et al., 2012, Garcia-Ruiz et al., 2010). *A. thaliana* plants deficient in core RNA silencing pathway genes display demethylation of the genome, hypersusceptibility to geminivirus infection and do not recover from infection with suppressor defective viruses (Raja et al., 2014, Raja et al., 2008). RNA-directed DNA methylation (RdDM) of the viral DNA genome is activated by DCL3 processing of double stranded RNAs into 24 nucleotide small RNAs (sRNA) that associate with AGO4 effector complex and trigger antiviral defense response (Castel and Martienssen, 2013, Matzke and Mosher, 2014). Therefore, recovery from geminivirus infection may suggest involvement of 24 nt sRNAs that direct virus genome methylation. However, there are no reports presenting its direct evidence in recovery from cassava mosaic geminiviruses (Rogans et al., 2016).

We report here characterization of the recovery phenomenon observed in CMD-infected cassava genotypes known to carry CMD1 (TMS 30572), CMD2 (TME 3, Oko-iyawo, TME 204) and CMD3 (TMS 97/2205) type resistance. Analysis of viral nucleic acids and deep sequencing of siRNAs involved in symptomatic and recovered leaves is described along with their relationships with CMD resistance.

## 2 Materials and methods

### 2.1 Plant materials and growth conditions

Cassava genotypes TME 3, Oko-iyawo, TME 7, TME 14 and TME 204 (CMD2 types), TMS 30572 and TMS 30001 (CMD1 types), TMS 97/2205 and TMS 98/0505 (CMD3 types), 60444 and Ebwanatereka (CMD susceptible) were selected for this study. Experimental plants were established in tissue culture and multiplied as described by Taylor et al. (2012). Two-week-old micropropagated plants were established and maintained in the greenhouse as described by Beyene et al. (2015). Though somatic embryogenesis has been demonstrated to induce loss of function in CMD2 resistance (Beyene et al., 2015), clonal micropropagation has no effect on CMD2 mediated resistance. Therefore, experimental materials were multiplied through tissue culture to generate uniform starting but not subjected to embryogenesis.

### 2.2 Cassava geminivirus inoculation

Infectious clones of two cassava mosaic virus species: *African cassava mosaic virus* Cameroon strain (ACMV-CM), NCBI accession numbers AF112352 and AF112353; and a Kenyan strain of *East African cassava mosaic virus* (EACMV-KE2) isolate K201, NCBI accession numbers AJ717541 and AJ704953, were used in the study. DNA A and DNA B genomic components were cloned from head to tail as partial tandem dimers, previously described by Patil and Fauquet (2010), and propagated in *Escherichia coli* (DH5α). Virus inoculation was performed according to the method described by Beyene et al. (2015). Eight plants per genotype were inoculated with each virus species and experiments were performed twice.

### 2.3 Assessment of *Cassava mosaic* disease in the greenhouse

Inoculated plants were monitored for development of disease symptoms starting five days post inoculation (dpi) and thereafter once per week for nine weeks. The proportion of plants showing CMD symptoms on the three uppermost newly opened leaves was expressed as the percentage of total number of plants inoculated. Disease severity was scored visually using a scale of 0–5 whereby 0 represents no disease symptoms and 5 represents severe mosaic with complete leaf curling as described by Beyene et al. (2015). In order to determine whether symptom attenuation was associated with clearance of viral particles, shoots were cut back to 10 cm above soil level at 75 dpi and all leaves removed. Two weeks after cutback, the new shoot growth was scored for disease symptoms as described by Beyene et al. (2015).

### 2.4 Total nucleic acid extraction

Total nucleic acid was extracted from newly opened leaves using cetyltrimethylammonium bromide (CTAB) method (Doyle and Doyle, 1990). Sampling was performed at 12, 20, 35, 45 and 65 dpi. After extraction, DNA samples were treated with 8 μl DNase free ribonuclease according to the manufacturer’s instructions (Roche, IN, USA), followed by chloroform extraction and ethanol precipitation. RNA samples were subjected to RNase-free DNase 1 (Ambion, Carlsbad, CA, USA) treatment at 37 °C for 30 min. DNA and RNA samples were quantified on a NanoDrop 2000c spectrophotometer (Thermo Scientific, DE, USA).

### 2.5 Southern blot analysis for virus DNA

Five micrograms of total genomic DNA were fractionated on a 1% (w/v) agarose gel at 34 V overnight (ca. 12 hrs). The DNA was transferred onto a positively charged nylon membrane (GE Healthcare, Piscataway, NJ, USA) using 20× SSC for at least 8 hrs and UV crosslinked (Green and Sambrook, 2012). Probes for virus detection were PCR amplified using the primers listed in Supplementary Table 1, which targeted the replicase (AC1) and movement protein (BC1) genes of each virus species. Probes were labelled with use of a digoxigenin (DIG) DNA labelling kit (Roche). The subsequent steps were performed as described by Beyene et al. (2015).

### 2.6 PCR detection of cassava mosaic geminiviruses

Detection of EACMV KE2 (K201) and ACMV-CM was performed in systemically infected leaves by PCR. Primers (Supplementary Table 1) were designed to target the virus replicase (*AC1*) and movement protein (*BC1*) genes specific to each virus species. The PCR was performed as follows: 95 °C for 3 min, followed by 35 cycles at 95 °C for 40 s, 58 °C for 30 s, 68 °C for 1 min and final extension at 68 °C for 10 min. The fragment sizes were confirmed on a 1% (w/v) agarose gel. The gel image was acquired and analyzed using Molecular Imager^®^ Gel Doc™ XR documentation system with Image Lab software (Bio-Rad, Hercules, CA).

### 2.7 Quantification of virus titer by real-time quantitative PCR

The virus titre of EACMV KE2 (K201) was assessed by real-time quantitative PCR (qRT-PCR) at full disease establishment (20 dpi) and the recovery phase (65 dpi). Sample DNA was standardized to a concentration of 4.5 ng/μl and 10 μl subjected to SYBR Green I chemistry as described by Ogwok et al. (2015). Amplification was performed using the following conditions: 95 °C for 3 min, followed by 40 cycles at 95 °C for 10 s and 60 °C for 30 s. Cytochrome c oxidase (COX) (Adams et al., 2013) was used as reference gene. The primers used for this experiment are listed in Supplementary Table 1. Three biological replicates for each sample were replicated into two technical replicates for qRT-PCR analysis.

### 2.8 Northern blot analysis for detection of virus-derived small RNAs

Low molecular weight RNA molecules were separated by Northern blotting of 30 μg total RNA on 15% Criterion TBE-Urea polyacrylamide gel (Bio-Rad, Hercules, CA, USA) at 100 V for 2 h and vsRNAs were detected (Patil et al., 2015c). Probes derived from AC1/AC2 region were amplified with primers described in Supplementary Table 1 and *in vitro* transcribed with DIG RNA labelling kit SP6/T7 (Roche, Indianapolis, IN, USA).

### 2.9 Construction of a small RNA library and sequencing

Double tagged cDNA libraries were prepared from 5 μg of total RNA obtained from EACMV KE2 (K201) infected and mock inoculated cassava plants at 20, 35 and 45 dpi. Small RNAs were sequentially ligated to adenylated NEBNext 3′ and 5′ SR adaptors for Illumina 5′-rAppAGATCGGAAGAGCACACGTCT-NH2-3′ and 5′- rGrUrUrCrArGrArGrUrUrCrUrArCrArGrUrCrCrGrArCrGrArUrC-3′. Adaptor ligated small RNA libraries were reverse transcribed into cDNA with ProtoScript II reverse transcriptase followed by PCR amplification with Illumina index primers. Size selection was performed on 6% polyacrylamide gel (Invitrogen, Carlsbad, CA, USA), whereby 140–150 nucleotide bands corresponding to adapter-ligated small RNAs of sizes 21–30 nucleotides were isolated to generate fragments for Hiseq Illumina sequencing.

### 2.10 Analysis of small RNA sequences

Samples were sent to Genome Technology Access Center (GTAC) at Washington University in St. Louis for quality check on Agilent Bioanalyzer and sequencing on Illumina HiSeq 2500 platform using 1 × 50 single-end read protocol. Raw sequencing data was demultiplexed by QIIME (Caporaso et al., 2010) and sequence reads with a quality score of 19 and below were excluded. Adapter sequences were trimmed by Cutadapt (Martin, 2011) and sequence reads in the size range of 21 to 24 nt selected for downstream analysis. Total reads plus collapsed reads (unique reads obtained from total reads by fastx_collapser- a software in FASTX-TOOLKIT) were mapped to the reference genome by Bowtie (Langmead et al., 2009). Mapped reads data was converted to statistical data by BEDTools (Quinlan and Hall, 2010) and all outputs graphically presented by the shell scripts provided (Fahlgren et al., 2009).

### 2.11 Real-time quantitative PCR of cassava specific dicer like protein genes

Cassava orthologous to *A. thaliana* DCLs was retrieved from the cassava reference genome version 6.1 database (http://phytozome.jgi.doe.gov/pz/portal.html). The nucleotide sequences were imported to MEGA6.0 and multiple alignments performed with respective DCL retrieved from *A. thaliana.* A phylogenetic tree was constructed using the near-neighbor joining method. Bootstrap analysis was performed with 500 replicates to test the strength of the nodes. Primers for DCL qRT-PCR are described in Supplementary Table 1. The transcript levels of DCLs were quantified using qRT-PCR as described by Ogwok et al. (2015).

## 3 Results

### 3.1 Cassava genotypes respond differently to infection by ACMV-CM and EACMV KE2 (K201), respectively

**Fig. 1:**
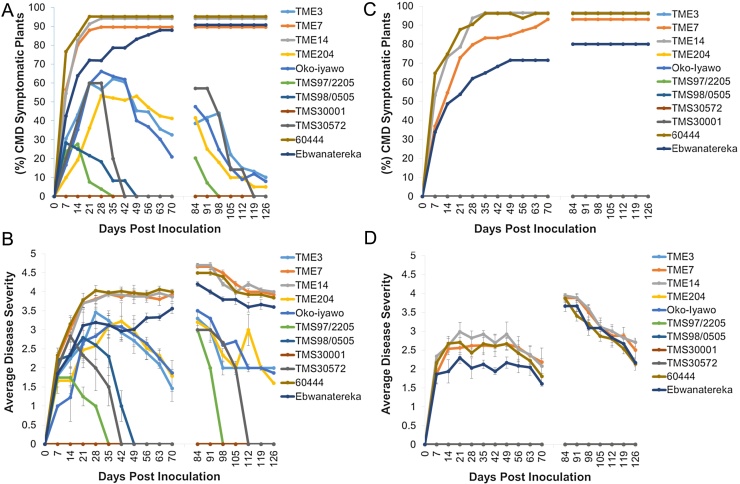
Response of cassava genotypes to infection with ACMV-CM and EACMV KE2 (K201) respectively. The histogram represents; (A) Number of plants that developed systemic symptoms after inoculation with EACMV-KE2 [K201] expressed as percentage, (B) Symptom severity induced by systemic infection with EACMV-KE2 [K201]. (C) Percentage of plants that developed systemic symptoms after inoculation with ACMV-CM. (D) Mild to severe symptoms induced by inoculation with ACMV-CM.

**Fig. 2:**
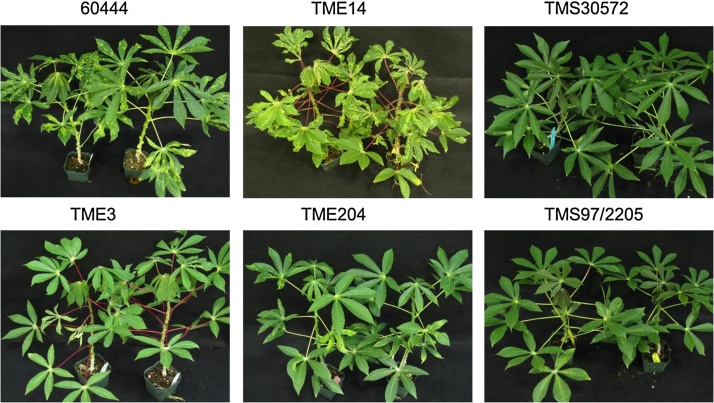
CMD symptom phenotype in cassava genotypes inoculated with EACMV KE2 (K201) at 65 dpi. Under greenhouse conditions, inoculation of EACMV-KE2 [K201] (AJ704953; AJ717541) into cassava genotypes induced severe systemic symptoms. However, over time the new systemic leaves of CMD resistant cassava genotypes showed markedly reduced symptoms and eventually became asymptomatic at 65 dpi.

Mosaic symptoms were first observed five days post inoculation (dpi) with the highest percentage of symptomatic plants across all genotypes recorded between 20 and 35 dpi (Fig. 1, Fig. 2). Varying levels of susceptibility to ACMV-CM and EACMV KE2 (K201) were recorded among the genotypes. Cassava genotypes carrying CMD resistant loci CMD1, CMD2 and CMD3 respectively showed high resistance to inoculation with ACMV-CM with no obvious ACMV-CM induced symptoms observed on their leaves (Figs. 1C and D). ACMV-CM viral DNA was PCR-detectable only in young systemically infected leaves at 7 dpi but not at 20 dpi in these CMD resistant genotypes (Supplementary Fig. 1). Conversely, in the susceptible cultivars 60444 and Ebwanatereka, 60–100% of plants inoculated with ACMV-CM developed systemic symptoms (Fig. 1C), with average severity of 2 to 3 (Fig. 1D). Viral DNA remained detectable by both PCR and Southern blot analysis in young leaves of susceptible cultivars throughout the experimental period (Supplementary Fig. 1A). Although cassava genotypes TME 7 and TME 14 were previously described as CMD resistant and shown to be derived from the same gene pool as other West African landraces carrying CMD2 locus (Lokko et al., 2005, Okogbenin et al., 2012, Rabbi et al., 2014), data presented here shows that the accessions studied here were susceptible to both ACMV-CM and EACMV KE2 (K201) (Fig. 1, Fig. 2). This was in contrast to other CMD2 types (TME 3 and Oko-iyawo) that did not develop symptoms following infection with ACMV-CM and showed recovery following infection with EACMV KE2 (K201) (Fig. 1, Fig. 2).

**Fig. 3:**
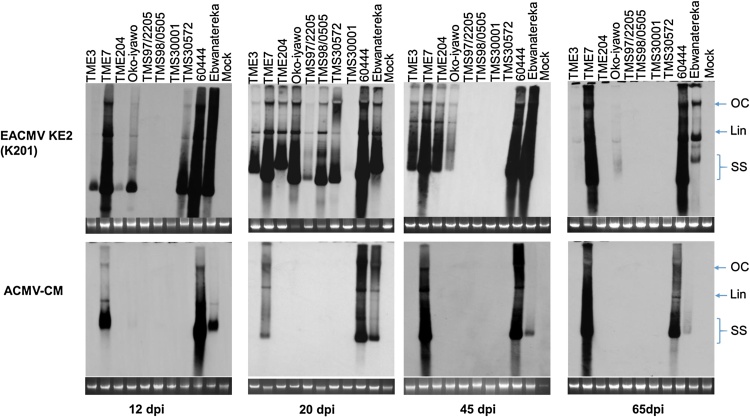
Detection of **v**iral DNA titer in different cassava genotypes using Southern blot. Five micrograms of cassava genomic DNA infected with EACMV KE2 (K201) and ACMV-CM were separated on 1% (w/v) agarose gel and transferred on positively charged nitrocellulose membrane. The membranes were hybridized with cocktail of DIG labelled probes derived from AC1 and BC1 genes. Different viral DNA conformations (open circular, linear and single stranded) are indicated on the blot. Differential virus titers were observed with peak accumulation of EACMV K201 (E201) observed at 20 dpi in resistant genotypes followed by complete clearance of DNA at 65 dpi. Susceptible genotypes accumulated high virus titer with the exception of Ebwanatereka that showed ACMV CM DNA reduction beginning 45 dpi.

Plants of all genotypes inoculated with EACMV KE2 (K201) developed more severe CMD symptoms (severity score up to 4), compared to those challenged with ACMV-CM (severity score up to 3) at full disease establishment (Fig. 1B and D). EACMV KE2 (K201)- induced symptoms were observed beginning 12 dpi in CMD2-type and susceptible cultivars with 50 to 100% of total plants developing an average symptom severity of 3 to 4 by 20–35 dpi (Fig. 1A and B). The CMD3-type cultivars TMS 97/2205 and TMS 98/0505, and the CMD1-type cultivar TMS 30572, were the most resistant to EACMV KE2 (K201) with less than 20% of plants inoculated developing average symptom severity of 2 by 20 dpi (Fig. 1A and B). Systemic symptoms induced by EACMV KE2 (K201) on TMS 97/2205 and TMS 98/0505 were restricted to no more than two leaves above the inoculation site (Fig. 2). By 49 dpi, all plants of cultivars carrying CMD1 and CMD3 resistance had produced asymptomatic new leaves. In contrast, CMD2-type genotypes showed partial recovery and continued to display mild CMD symptoms (score 2) on 20–40% of plants at 65 dpi (Figs. 1 B and 2). Upon complete CMD symptom recovery in CMD1, CMD2 and CMD3 cultivars, viral DNA could not be detected within asymptomatic young leaves at 65 dpi, either by PCR or Southern blot analysis (Fig. 3 and Supplementary Fig. 1). In susceptible genotypes, up to 100% of plants inoculated with EACMV KE2 (K201) developed average symptom severity of 4 and exhibited high viral DNA loads throughout the observation period (Figs. 1 A, 2 and 3). These plants displayed persistent symptoms with no sign of recovery from infection (Figs. 1 B, 2 and Supplementary Fig. 1).

In order to verify whether recovery from CMD was a temporary or permanent phenomenon, stems of all inoculated and control plants were cut back to 10 cm above soil level at 75 dpi and all leaves removed. Shoot tissues were allowed to regrow and newly formed leaves assessed for presence and severity of CMD symptoms. A flush of severe symptoms was observed on new growth of plants of susceptible cassava genotypes challenged with ACMV-CM, reaching an average severity score of 4 (Fig. 1D). However, new growth of CMD1, CMD2 and CMD3-type genotypes was devoid of ACMV-CM symptoms (Fig. 1D). A wide variation in disease symptoms was observed after cutback among cassava genotypes inoculated with EACMV KE2 (K201) (Fig. 1B). Susceptible plants showed severe symptoms characterized by yellow mosaic on leaves, leaf curling, and reduced leaf size with severity scores reaching 4 (Fig. 1B, Supplementary Fig. 2). Twenty-five percent of CMD3-type resistant plants developed mild symptoms compared with 40% and 60% of plants from TMS 30572 (CMD1) and CMD2 resistant types, respectively (Fig. 1A). CMD symptoms seen on TMS 30572 (CMD1), CMD2 and CMD3 cassava genotypes were observed on the first few new leaves with the plants then undergoing recovery from disease to become free of symptoms (Fig. 1B, Supplementary Fig. 2). Cassava genotype TMS 30001 showed highest resistance to both ACMV CM and EACMV KE2 (K201) infection (Figs. 1 A,B and 3). However, plants of TMS 30001 were highly susceptible to red spider mites leading to defoliation and stunting. These plants did not recover from mite damage following application of pesticides.

### 3.2 Detection of virus DNA in infected cassava genotypes

Systemically infected cassava plants were analyzed for viral DNA accumulation at 12, 20, 45 and 65 dpi by Southern blotting using a cocktail of DIG labelled probes for detection of *AC1* and *BC1* virus genes (Fig. 3). Different geminivirus DNA conformations including open circular, linear and single stranded were detected on these blots (Fig. 3). Cassava genotypes carrying CMD1, CMD2 or CMD3 type resistance did not accumulate detectable levels of ACMV-CM. On the contrary, ACMV-CM virus DNA accumulated to significant levels in the susceptible genotypes 60444, Ebwanatereka, TME 7 and TME 14. These cultivars displayed minimal recovery from disease and no obvious change in virus load over time, with the exception of Ebwanatereka. This cultivar showed a reduction of ACMV-CM viral DNA by 45 dpi, correlating with lower ACMV-CM symptomatic plants compared to TME 7 and 60444 (Fig. 1C and D).

Viral DNA of EACMV KE2 (K201) was detectable starting at 12 dpi with susceptible genotypes accumulating the highest viral DNA load (Fig. 3). Between 20 and 35 dpi, similar levels of viral DNA were observed in susceptible and CMD2 genotypes, while CMD1 and CMD3 genotypes accumulated lower levels of viral DNA (Fig. 1, Fig. 3). Virus titer was assessed by qRT-PCR at 20 dpi and 65 dpi (Supplementary Fig. 1B). Virus titer accumulated by plants of 60444 at 20 dpi was three times greater than that detected in plants of CMD2-types TME 3, TME 204 and Oko-iyawo, and the CMD1-type TMS 30572, and seven times greater than in the CMD3-type TMS 97/2205 (Supplementary Fig. 1B). After the onset of recovery in CMD1, CMD2 and CMD3 genotypes, viral DNA load was significantly reduced, becoming undetectable in asymptomatic young leaves by 65 dpi (Fig. 3D, Supplementary Fig. 1B). Conversely, susceptible genotypes showed consistently high concentration of virus DNA associated with absence of recovery from CMD symptoms when infected with EACMV KE2 (K201) (Figs. 1 B, 3 A-D and Supplementary Fig. 1B).

### 3.3 Northern blot analyses of virus derived small RNA in cassava genotypes

Virus infection in plants triggers production of virus derived small RNAs (vsRNAs) that potentially direct antiviral defense. Presence of vsRNAs within young leaves systemically infected with EACMV KE2 (K201) and ACMV-CM was assessed by Northern blot hybridization with virus specific probes derived from AC1/AC2. ACMV-CM derived vsRNA was not detected by Northern blot hybridization within tissues of the CMD resistant cultivars TME 3, TME 204, Oko-iyawo, TMS 30572 and TMS 97/2205 throughout the experimental period (Supplementary Fig. 3). Accumulation of vsRNA was detectable as early as 12 dpi in samples collected from symptomatic tissues, and corresponded with the onset of CMD symptoms for both virus species (Fig. 1, and Supplementary Fig. 3). CMD susceptible genotypes accumulated vsRNAs from the onset of symptom development (12 dpi) with no significant change in quantity observed for the remainder of the experimental period (Supplementary Fig. 3). Among the susceptible genotypes, Ebwanatereka accumulated lower quantities of ACMV-CM vsRNAs compared with 60444 and TME 7.

CMD2-type and susceptible cultivars accumulated high amounts of EACMV KE2 (K201) derived vsRNAs at 20–35 dpi, which correlated with high disease severity at that time (Fig. 1B and Supplementary Fig. 3). However, by 45 dpi, EACMV KE (K201) vsRNAs were significantly reduced in CMD2 genotypes corresponding with the symptom recovery phase. At 20 dpi, CMD1 and CMD3 types accumulated the lowest amount of EACMV KE (K201) vsRNAs. Based on quantification of siRNA signal intensity, this quantity was three times less than that accumulated in CMD2 types and susceptible cassava plants.

### 3.4 Deep sequencing of small RNAs in ACMV and EACMV KE2 (K201) infected cassava genotypes

Profiles of vsRNAs produced in cassava genotypes during infection with EACMV KE2 (K201) and ACMV-CM were obtained by Illumina Solexa sequencing of total small RNAs isolated from systemically infected leaves. Cassava genotypes showed a differential response in the amount of vsRNAs accumulated in response to infection with the two virus species (Table 1, Supplementary Fig. 3). The total reads mapping to EACMV KE2 (K201) were between 55,236 and 608,476 while those specific to ACMV-CM were between 30,236 and 64,084. The reads obtained from non-infected controls of TME 3, TME 204 and TMS 30572 but mapped to the virus genome ranged from 142 to 178 (Table 1). The results indicate that upon infection with a virulent isolate of a severe CMG species such as EACMV KE2 (K201), cassava genotypes accumulate two to eight times more vsRNAs than when infected with a less virulent CMG species such as ACMV-CM. From both Southern blot and qRT-PCR analysis, cassava genotypes infected with EACMV KE2 (K201) were found to accumulate high virus DNA titer compared with those infected with ACMV-CM (Figs and Supplementary Fig. 2). Virus titer was positively correlated with vsRNAs using Southern blot, deep sequencing and Northern blot (Supplementary Fig. 3 and Table 1).

**Fig. 4:**
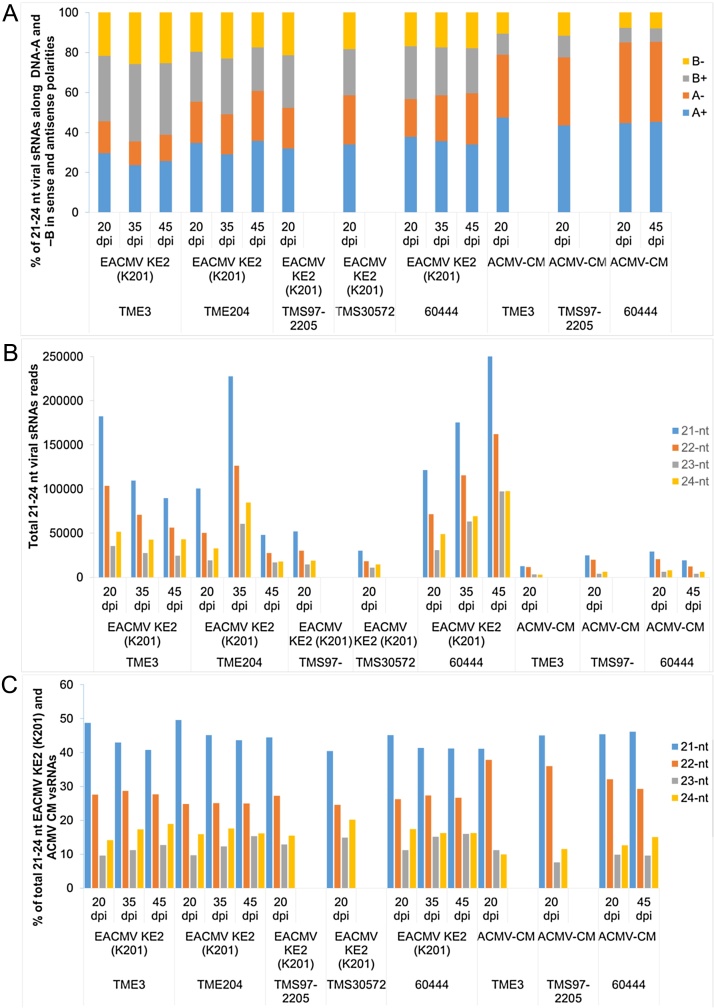
Illumina deep sequencing of small RNAs in cassava infected with EACMV KE2 (K201) and ACMV-CM, respectively. The graphs show: (A), proportions of EACMV KE2 (K201) and ACMV-CM vsRNAs respectively derived from DNA A and DNA B components in sense and antisense orientation; (B) total vsRNAs of size classes 21–24 nt vsRNAs mapped to the virus genome with zero mismatches; (C) 21–24 nt vsRNAs expressed as percentage of the total sRNAs.

Cassava genotypes 60444, TME 204 and TME 3 were challenged with EACMV KE2 (K201) and three individual plants sampled at three time points, depicting disease establishment (20 dpi), maximum disease severity (35 dpi) and the recovery phase (45 dpi). EACMV KE2 (K201) derived siRNA was most abundant in 60444 and TME 3 ranging from 23% to 38% of total siRNA across the three different time points, whereas in TME 204, EACMV KE2 (K201) derived siRNA ranged from 18% to 22% (Table 1). The proportion of EACMV KE2 (K201) vsRNAs mapping to DNA A and B components differed in the different genotypes (Fig. 4A). In cultivar 60444, EACMV KE2 (K201) vsRNAs derived from DNA B were 19% of the total mapping reads compared with 13% reads mapping to the DNA A component. For TME 3, DNA A derived vsRNA was 16% and DNA B 12%, whereas in TME 204, both DNA A and DNA B derived siRNA were in almost equal proportions at 10% and 9%, respectively (Table 1).

Leaf samples were collected only at 20 dpi from cultivars TMS 97/2205 and TMS 30572 and then challenged with EACMV KE2 (K201) for the study of siRNA. After this time point, these plants had fully recovered from virus infection (Fig. 1A) and geminivirus-derived siRNA was not detectable on Northern blots (Supplementary Fig. 3). In TMS 97/2205, approximately 10% of the total siRNA present in the plant tissue was derived from EACMV KE2 (K201) whereas in TMS 30572 this value was about 6%. siRNAs derived from the DNA A and DNA B components were seen in almost equal proportions in both of these cultivars. Similarly for cassava genotypes TMS 97/2205, TME 3 and 60444 challenged with ACMV-CM, leaf samples were collected for siRNA study at only the first time point (20 dpi). About 7% of the siRNA mapped to ACMV-CM in genotypes TMS 97/2205 and 60444, whereas in TME 3 only 4% of siRNA was derived from ACMV-CM. In all three genotypes, siRNA mapping to the DNA A component was >75% of the total mapping reads compared to <20% mapping to the DNA B component (Fig. 4A). The number of siRNAs from mock-inoculated genotypes mapping to the sequences of EACMV KE2 (K201) and ACMV-CM amounted to 0.02% (Table 1).

EACMV KE2 (K201) vsRNAs exhibited bias in strand polarity, whereby >55% of total mapped reads originated from the sense orientation for both genomic components (Fig. 4A). A shift in vsRNAs was observed in plants inoculated with ACMV-CM, whereby CMD susceptible genotype 60444 had almost equivalent amounts of positive (52%) and negative (48%) vsRNAs derived from both DNA A and B, respectively. In contrast, the CMD resistant genotypes TME 3, TME 204 and TMS 97/2205 had proportionally higher (55–59%) vsRNAs obtained from DNA A in the sense direction and equally distributed ACMV-CM vsRNAs in sense and antisense direction along DNA B. A polarity distribution ratio of almost one in the sense and antisense vsRNAs implies that dsRNA precursors are processed from both positive and negative virus strands to generate small RNAs.

In all CMD infected cassava genotypes, small RNA size classes of vsRNAs 21, 22, 23 and 24 nt in size spanning the entire virus genome in sense and antisense orientations were identified (Fig. 4B). The most predominant vsRNAs across all cassava genotypes for the two virus species were 21 nt (up to 50%) followed by 22 nt (up to 25%), while 24 nt sized vsRNAs comprised only 15% of the total vsRNAs.

### 3.5 Distribution of vsRNAs along the cassava-infecting geminiviruses genome

**Fig. 5:**
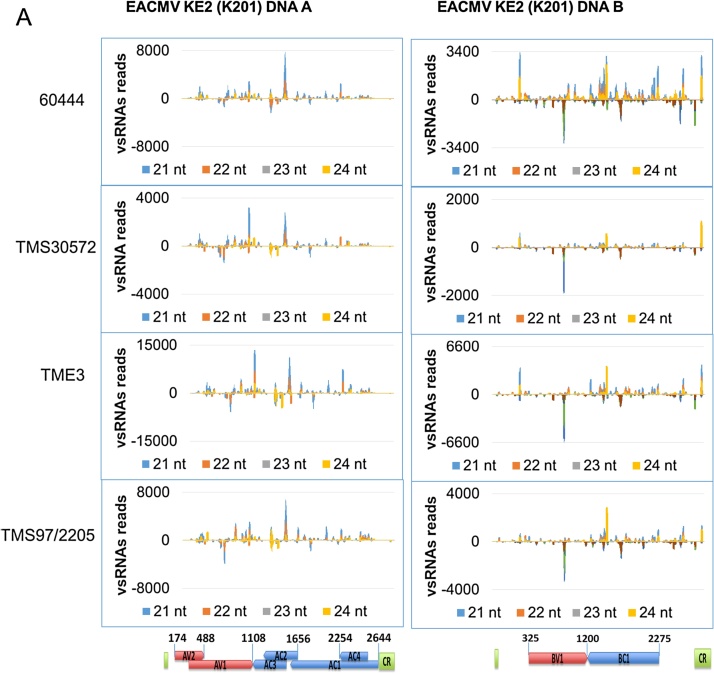

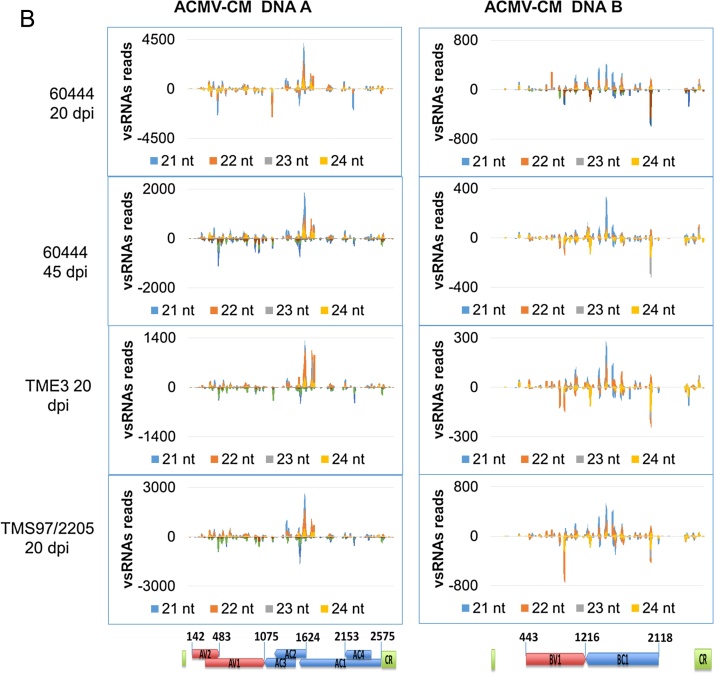
Genome wide distribution of virus derived small RNA in positive and negative polarities. (A) Small RNA reads mapped along the genome of EACMV KE2 (K201). (B) Small RNA reads mapped along the genome of ACMV-CM.

The pattern of vsRNA distribution along the virus genome was assessed according to their dichotomy and genomic position. From the analysis of redundant sRNA reads, several sharp peaks were identified unevenly distributed across the virus genome (Fig. 5). Hotspots of 21 and 22 nt EACMV KE2 (K201) vsRNAs were more prominent in the sense orientation within the coding regions of AV2 (800–910), AC2/AC3 (1300–1400), BC1 (1400–1500), IRB (2700) and an antisense peak corresponding to BV1 (900–1000). Along the EACMV KE2 (K201) DNA A genome, 24 nt vsRNAs were homogenously distributed, but two major 24 nt hotspots were identified on the DNA B genome at the promoter region and the BC1 gene on the positive strand. A few strand gaps of less than 20 nucleotides in EACMV KE2 (K201) were identified in the 5′ and 3′ end of the virus genome corresponding to the non-coding intergenic region. However, these gaps were covered on the opposite strand.

On the ACMV-CM genome, the major small RNA peaks were observed in sense orientation on DNA A (nt-1361-1701) and DNA B (nt-1361-1701) and on antisense orientation on DNA B (nt-851-1021 and nt-2011) (Fig. 5B). Alignment of small RNAs and ACMV-CM nucleotide sequences revealed poor coverage in intergenic regions as well as some transcription unit regions. The nucleotide positions where gaps were found included: 1–100, 1066–1188, 1715–1862, 1976–2078, 2350–2373 and 2659–2748 for ACMV-CM DNA A; and 1–450 and 2156–2546 for ACMV-CM DNA B (Fig. 5B).

The density of vsRNAs was evaluated to identify the abundance of small RNAs relative to ORF and/or overlapping regions. High abundance of small RNAs was identified in complementary and virion sense transcriptional units of both DNA A and DNA B, respectively for the two viruses, with positive and negative reads represented equally (Fig. 5 and Supplementary Fig. 4). Low density of vsRNA reads from intergenic regions of the DNA A component for both ACMV-CM and EACMV KE2 (K201) were observed, pointing towards preferential targeting of the virus genome by DCLs. vsRNA accumulation on each virus transcriptional unit differed between cassava genotypes (Supplementary Fig. 4). Overall, 60444 accumulated highest EACMV KE2 (K201) vsRNAs corresponding to BC1, BV1 and IRB whereas TMS 30572, TME 3 and TMS 97/2205 accumulated similar amounts of vsRNA on complementary and virion sense transcriptional units. However, in plants of 60444, TMS 30572, TME 3 and TMS 97/2205 infected with ACMV-CM, predominant vsRNAs were derived from transcriptional units of DNA A.

The GC content of the CMG genome may influence its targeting by host DCLs for antiviral silencing. The ACMV-CM genome contains 45% GC in DNA A and 41% in DNA B while EACMV KE2 (K201) contains 46% GC in DNA A and 43% in DNA B (Supplementary Table 2). For both virus species, AC1/AC2, AC4, AV2 and IR right; and, for EACMV KE2 (K201), BC1, BV1 and IR left, are GC rich at greater than 45% (Supplementary Table 2). Genome-wide distribution of vsRNAs along the virus genome identified various hotspots between nucleotide position 900–2000 for DNA A and 900–1801, corresponding to high GC regions (Fig. 5A and B). However, low reads were observed in other GC-rich regions such as DNA A IR right for both viruses, indicating a strategy by cassava-infecting geminiviruses to limit siRNAs targeting promoter regions for transcriptional silencing.

### 3.6 Abundance of vsRNA 5′-terminal nucleotide

**Fig. 6:**
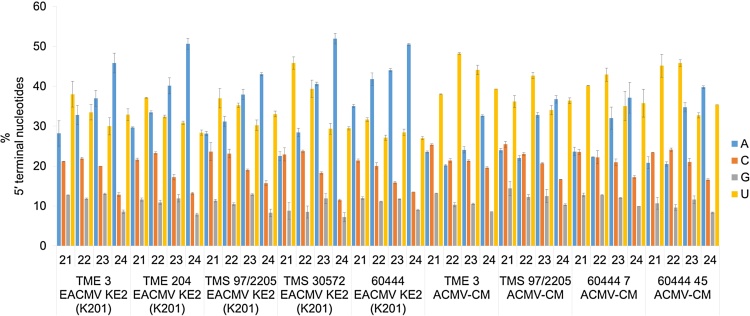
Relative frequency 5′-terminal nucleotides of 21–24 nt species of vsRNAs.

To elucidate the potential interaction of EACMV KE2 (K201) and ACMV-CM vsRNAs with distinct cassava AGO complexes, the 5′-terminal nucleotide composition of different-size species was analyzed (Fig. 6). Overall, for the two CMG species, there was a strong bias for adenosine (A), uridine (U), cytosine (C) and tendency to avoid guanidine (G) as 5′-terminal nucleotide of vsRNA sense polarity regardless of cassava genotype analyzed. vsRNA reads of 21- 22- and 23-nt in length showed dominant A or U (28–40%), respectively, at their 5′-terminal position indicating potential loading into AGO1 and AGO2, while 24-nt vsRNAs presented A as the most abundant 5′-terminal nucleotide (43–50%), indicative of high affinity to AGO4 (Fig. 6). Noticeably, ACMV CM derived vsRNAs mostly contained significantly (p ≤ 0.05) higher U while EACMV KE2 (K201) vsRNAs contained significantly (p ≤ 0.05) more A as the most abundant first nucleotide (Fig. 6).

### 3.7 Differential activity of DCLs in virus susceptibility among different cassava plants infected by geminiviruses

**Fig. 7:**
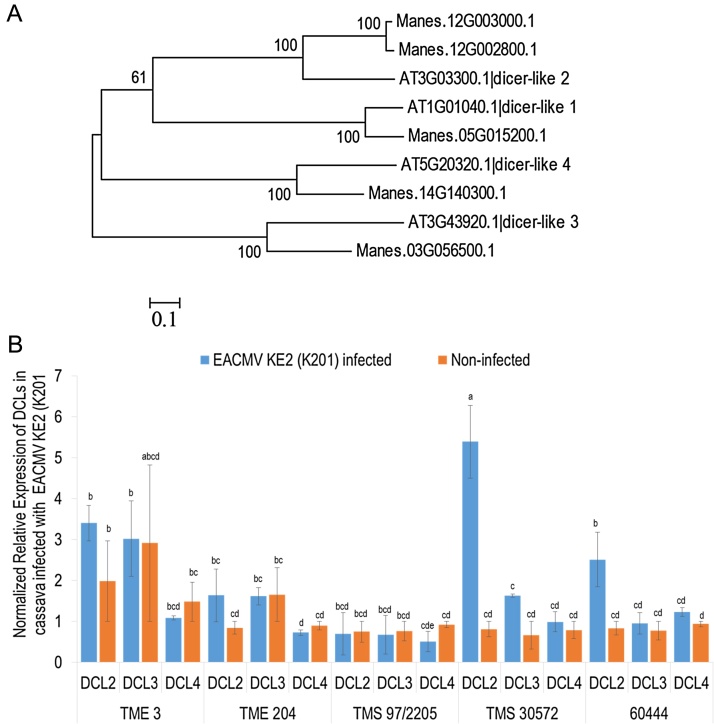
Transcript quantification of homologues of *ManesDCLs* in cassava genotypes infected with EACMV KE2 (K201) at 32 dpi.

Each of the four Arabidopsis DCL orthologs were identified from the cassava genome as follows: DCL1 (Manes.05G015200.1); DCL2 (Manes.12G002800.1 and Manes.12G003000.1); DCL3 (Manes.03G056500.1); and DCL4 (Manes.14G140300.1) (Fig. 7A). Transcript levels of three DCLs that mediate antiviral defense were assessed by qRT-PCR in plants infected with EACMV KE2 (K201) at 35 dpi. In cassava genotypes TME 3, TME 204, TMS 30572 and 60444, the expression of DCL2 was found to be upregulated in plants infected with EACMV KE2 (K201) compared with mock inoculated plants. However, no altered expression of DCL3 or DCL4 was detected in any of the cassava genotypes studied (Fig. 7B).

## 4 Discussion

Three sources of natural resistance to CMD have been characterized in cassava (Okogbenin et al., 2012, Rabbi et al., 2014). Data presented here shows high resistance in response to challenge with infectious CMG clones in CMD1, CMD2 and CMD3 resistance type cultivars (Fig. 1, Fig. 2). Quantitative CMD resistance derived from a cross between *M. glaziovii* × *M. esculenta* has been reported in TMS 30001 and TMS 30572 (Jennings, 1976). These genotypes showed immunity to infection by ACMV-CM in the present study (Fig. 1, Fig. 2). However, differential response to EACMV KE2 (K201) infection was observed. TMS 30001 plants remained asymptomatic, whereas TMS 30572 plants developed CMD symptoms followed by complete recovery (Fig. 1, Fig. 3). CMD3-type cassava cultivars TMS 97/2205 and TMS 98/0505 (Dixon et al., 2010, Okogbenin et al., 2012) showed highest resistance to CMD and rapid recovery from EACMV KE2 (K201) infection in the greenhouse (Fig. 1). This correlates with reports from field studies that indicate low CMD incidence and mild symptoms in TMS 97/2205 and TMS 98/0505. TMS 97/2205 is derived from a cross between TME 6 (CMD2 genotype) and TMS 30572 (CMD1 genotype). Therefore, based on Mendelian inheritance, high CMD resistance may be attributable to synergistic interaction between CMD1 and CMD2 resistance mechanisms, in addition to the newly identified CMD3 locus (Okogbenin et al., 2012). Although previously described as CMD resistant (Fregene et al., 2000, Lokko et al., 2005), the specific clones of genotypes TME 7 and TME 14 held in collections at DDPSC were found to be susceptible to both ACMV-CM and EACMV KE2 (K201) (Fig. 1, Fig. 2). In contrast, Oko-iyawo and TME 3, both previously reported to be genetically similar to TME 7 and TME 14 (Rabbi et al., 2014), were found to be highly resistant to ACMV-CM and completely recovered from EACMV KE2 (K201) infection (Fig. 1, Fig. 2). Recently, breakdown of CMD2 mediated resistance was reported after somatic embryogenesis of CMD2 type cassava genotypes (Beyene et al., 2015). However, this phenomenon was not demonstrated in the CMD3 genotype TMS 98/0505 after passage through embryogenesis (Beyene et al., 2015). Data presented here showed 0–60% symptomatic plants of CMD1 and CMD3 genotypes with an average disease severity score of 3 compared with more than 60% symptomatic plants of CMD2 genotypes and average disease severity score 3.5 at maximum disease establishment (Fig. 1). CMD2 resistance is mediated by a single dominant gene that may easily be compromised as a result of stable genetic and epigenetic changes (Akano et al., 2002, Beyene et al., 2015). Data presented in this study showing high CMD resistance mediated by multiple genes in CMD1 and CMD3 genotypes supports the wisdom of recent efforts by different research groups to stack different genes for CMD resistance (Okogbenin et al., 2013).

The two species of CMGs evaluated in the present study differ in their pathogenicity. EACMV KE2 (201) induced disease in all cassava genotypes while ACMV-CM failed to induce symptoms in genotypes carrying innate CMD resistance mechanisms (Figs. 1–3). Virus encoded suppressors of gene silencing (VSR) have been implicated in virus pathogenicity (Csorba et al., 2015, Derrien et al., 2012, Garcia-Ruiz et al., 2015). Although the suppressors encoded by CMGs have not been well characterized, it is suggested that ACMV-CM *AC4* targets post-transcriptional gene silencing (PTGS) whereas EACMV and ICMV *AC2* potentially target both PTGS and transcriptional gene silencing (TGS) (Sun et al., 2015, Vanitharani et al., 2004). ICMV isolate ICMV-SG was recently shown to accumulate higher virus titer and greatly represses TGS in *N*. *benthamiana* compared to a less virulent isolate ICMV-Dha (Sun et al., 2015). Targeting PTGS and TGS by EACMV KE2 (K201) *AC2* may contribute to the high pathogenicity of the former as compared to ACMV-CM reported here. Further experiments are required to unravel the molecular mechanisms through which CMG suppressors of gene silencing subvert host antiviral defense.

Recovery from CMD infection was associated with reduction in viral DNA titer and vsRNA quantity in CMD1, CMD2 and CMD3 resistant types in a manner that reflected the visually scored disease curve as evidence of activation of potent defense mechanisms that clear systemic infection (Figs. 3, 4 and Supplementary Fig. 1). Highly symptomatic leaf tissues having the highest viral DNA load in the CMD2-type cultivar TME3 and susceptible cultivar 60444 accumulated the highest percentage (38%) of total siRNAs reads mapping to the viral genome. In contrast, in TMS 30572 (CMD1) and TMS 97/2205 (CMD3), mild symptoms were observed with associated low virus titer, and only 6–10% of total sRNAs reads mapping to the virus genome (Fig. 3 and Table 1). Onset and continuation of symptom recovery in CMD2 genotypes resulted in reduction of vsRNAs, while vsRNAs remained unchanged in 60444 (Fig. 3 and Table 1). Mild symptoms observed on new growth of fully recovered cassava plants of CMD1 and CMD2 genotypes after cutback suggests suppressed viral populations infecting few host cells (Fig. 1, Supplementary Fig. 2). Similar results were reported in pepper plants recovering from infection by pepper golden mosaic virus (PepGMV), suggesting interruption of plant defense mechanisms downstream of vsRNAs production (Rodriguez-Negrete et al., 2009). Methylation of the geminivirus DNA genome has been reported as a dominant defense mechanism that impedes viral replication and transcription, resulting in host recovery from infection (Butterbach et al., 2014, Raja et al., 2014, Sun et al., 2015). It is important that further investigations focus on determining whether targeted methylation of the viral genome is a functional component of the CMD1, CMD2 and CMD3 resistance mechanisms in cassava.

In *A. thaliana,* four DCL proteins act hierarchically and exhibit functional redundancy to actuate sRNA biogenesis (Deleris et al., 2006). DCL1 processes 21 nt miRNAs with limited antiviral response (Qu et al., 2008), while DCL2, DCL3 and DCL4 process long dsRNAs into 22, 24 and 21 nt sizes that mediate antiviral defense (Aregger et al., 2012, Blevins et al., 2006). In the present study, 22–37% of total sRNA reads recovered from 60444 were mapped to EACMV KE2 (K201), whereas TMS 30572 and TMS 97/2205 generated 5–20% of vsRNA reads (Table 1). Despite accumulation of lowest amounts of vsRNAs, TMS 30572 and TMS 97/2205 displayed highest resistance (symptom score <3) to cassava-infecting geminiviruses (Fig. 1). Data presented here contradict that of Chellappan et al. (2004) who reported low abundance of vsRNAs in highly symptomatic, and high vsRNAs in recovered leaves of cassava genotype 60444 and *N. benthamiana* infected with CMGs. Considering the small genome of cassava-infecting geminiviruses (2.8 kb) (Hanley-Bowdoin et al., 2013), the large fraction of vsRNAs (20–38%) produced by CMD infected cassava (Table 1) may reflect high virus replication/amplification rates as well as a strong response of the plant defense mechanism. Studies by Aregger et al. (2012) reported similar high abundance of total vsRNAs reads in Arabidopsis plants infected with *Cabbage leaf curl virus* (CabLCV). Differences in the relative accumulation of vsRNAs among cassava genotypes studied here may indicate varying proportions of infected cells in the tissues analyzed whereby in susceptible plants, cassava-infecting geminiviruses replicate to high copy numbers while innate and/or adaptive antiviral defense mechanisms limit virus replication in resistant genotypes to few cells.

Using blot based hybridization, Patil and Fauquet (2015a) demonstrated accumulation of high abundance of vsRNAs in *N. benthamiana* systemically infected with diverse species of CMGs. However, that study did not resolve vsRNAs based on their size classes. The highest proportion of EACMV KE2 (K201) vsRNAs originated from *AV1, AC1, AC3, BC1* and *BV1* whereas ACMV CM vsRNAs were mostly derived from *AV1, AV2, AC1* and *AC2,* indicating unique processing of vsRNAs from different CMGs (Supplementary Fig. 3). Different patterns of CMG siRNA distribution along the genome have been reported using reverse Northern blot (Patil and Fauquet, 2015a). Comparison of vsRNA distribution obtained from reverse-Northern hybridizations and deep sequencing may indicate substantial differences. Previous studies have demonstrated that methylated siRNAs are not amenable to cloning during library preparation but they are detectable using Northern blot hybridization (Raabe et al., 2014).

Across all the cassava genotypes infected with ACMV and EACMV-K201, the most predominant vsRNA species were seen as 21 nt (35–50%) followed by 22 nt (22–30%) and 24 nt (15–25%) (Fig. 4), suggesting presence of hierarchical action of DCL4, DCL2 and DCL3 in the biogenesis of CMG vsRNAs (Blevins et al., 2006, Deleris et al., 2006). CMG-derived vsRNA classes reported here are consistent with those described by Miozzi et al. (2013) for *Solanum lycopersicum* infected with the monopartite begomovirus tomato yellow leaf curl Sardinia virus (TYLCSV). Other studies have shown variation in size allocations of geminivirus-derived sRNAs in different host plants (Aregger et al., 2012, Blevins et al., 2006, Yang et al., 2011). All these reports suggest coordinated action of multiple DCLs in biogenesis of geminivirus vsRNAs.

EACMV KE2 (K201) and ACMV-CM vsRNAs were mapped and found to cover the entire DNA A and DNA B genomes in both sense and antisense polarities (Fig. 5), indicating POI II bidirectional transcription of the entire circular dsDNA (Pooggin, 2013, Rajeswaran and Pooggin, 2012). vsRNAs of sizes 21, 22, 23 and 24 nt localized within the same genomic regions that produced peaks (Fig. 5). The dense peaks were more prominent in genomic regions coding for overlapping transcripts and lowest on the intergenic region. vsRNA hotspots corresponded with highest abundant transcripts as reported by Patil et al. (2015b), possibly indicating regions within the virus genome that are preferentially targeted by DCLs. Similar distribution of vsRNA hotspots have been reported for TYLCCNV (Yang et al., 2011), CaLCuV (Aregger et al., 2012), *Wheat streak mosaic virus* (WSMV) and *Triticum mosaic virus* (TriMV) (Tatineni et al., 2014). Accumulation of 24 nt vsRNAs has been shown to enhance methylation of the intergenic region (Rodriguez-Negrete et al., 2009). Therefore, by limiting siRNAs in promoter regions, CMGs may have evolved a strategy for escaping TGS. vsRNAs derived from different cassava genotypes infected with CMGs showed similar patterns of size and distribution along the virus genome (Fig. 5). In agreement with the results present here, Rogans et al. (2016) demonstrated cassava genotypes TME3 and T200 systemically infected with SACMV displayed similar patterns of vsRNA distribution along the SACMV genome. These results indicate conserved processing of vsRNAs in different cassava genotypes but potentially different downstream processes of RNA silencing attributable to recovery from CMD infection.

Association of sRNAs and AGO proteins is mostly determined by 5′-terminal nucleotides and the structure of sRNA duplex (Garcia-Ruiz et al., 2015, Zhang et al., 2014). The majority of CMG vsRNAs of 21- and 22 nt size classes in the present study showed preference for 5′-A and 5′-U, respectively, an indication of involvement of AGO2 and AGO1 in antiviral defense (Fig. 6). The pool of 24 nt vsRNAs exhibited inclination to start with 5′-A (50%), suggesting loading into AGO4 complex (Fig. 6). Results presented here indicate involvement of multiple AGO complexes in defense against CMGs. For the two CMG species studied, ACMV-CM derived vsRNAs mostly contained significantly (p ≤ 0.05) higher U, while EACMV KE2 (K201) vsRNAs contained significantly (p ≤ 0.05) more A as the most abundant first nucleotide. Therefore, vsRNAs derived from ACMV-CM are preferentially loading into AGO1 while those derived from EACMV KE2 (K201) are preferentially associated with AGO2 and AGO4. Immunoprecipitation of AGO proteins from cassava genotypes infected with CMGs and deep sequencing will provide an insight into AGO-small RNA association and reveal sorting of CMG vsRNAs into diverse AGO.

Exploiting inherent resistance to CMGs remains the most important strategy to mitigate CMD pandemics. CMD1 and CMD2 types have been employed to mediate resistance to CMD for several decades (Okogbenin et al., 2013). However, recent reports describe breakdown of CMD2 resistance after passage of tissues through somatic embryogenesis (Beyene et al., 2015), indicating risks associated with over-reliance on a single strategy for disease resistance. The available transcriptome data from TME 3 (CMD2 type) infected with South African cassava mosaic virus (SACMV) fails to map differentially regulated genes to the CMD2 locus (Allie et al., 2014). Data presented here identify activation of defense mechanisms that effectively clear viral DNA from cassava genotypes carrying innate resistance to CMD. Cassava genotypes TMS 97/2205 and TMS 98/0505 showed robust resistance to the most pathogenic species of CMGs and are in line with current breeding efforts of incorporating multiple resistance types in breeding programs (Okogbenin et al., 2013). To understand the mechanisms of CMD resistance, more efforts should be focused on the role of epigenetics, and the refinement of existing cassava genome and transcriptome data.

## Conflict of interest

The authors have no conflict of interest to declare.

## Funding

This research was supported by the Bill and Melinda Gates Foundation; the United States Agency for International Development from the American people; and the Monsanto Fund. The sponsors had no role in study design, in the collection, analysis and interpretation of data; in the writing of the report; nor in the decision to submit the article for publication.

## Figures and Tables

**Table 1:** Average number of vsRNA reads derived from different cassava genotypes at various time points after infection with EACMV KE2 (K201) and ACMV-CM. The value in brackets represents percentage of reads mapping to viral genome with zero mismatches.

Cassava genotype	Virus species	Time point (dpi)	Total Clean reads	Total mapped reads	Mapped to DNA A Component	Mapped to DNA B Component
TME3	EACMV KE2 (K201)	20	1086892	372890 (33%)	211453 (19%)	161436 (14%)
TME3	EACMV KE2 (K201)	35	928091	250626 (25%)	133169 (14%)	117456 (11%)
TME3	EACMV KE2 (K201)	45	928700	213235 (17%)	133184 (11%)	80051 (6%)
TME204	EACMV KE2 (K201)	20	1044700	202728 (19%)	112935 (10%)	89793 (8%)
TME204	EACMV KE2 (K201)	35	2216538	499272 (19%)	251793 (9%)	247479 (10%)
TME204	EACMV KE2 (K201)	45	599230	109817 (18%)	55129 (9%)	54688 (9%)
TMS97/2205	EACMV KE2 (K201)	20	1129338	115616 (10%)	65027 (6%)	50589 (4%)
TMS30572	EACMV KE2 (K201)	20	982753	55236 (6%)	32615 (4%)	22620 (2%)
60444	EACMV KE2 (K201)	20	1009236	272925 (29%)	126951 (13%)	145974 (16%)
60444	EACMV KE2 (K201)	35	1124275	423335 (38%)	153082 (13%)	270253 (24%)
60444	EACMV KE2 (K201)	45	1879999	608476 (32%)	236798 (12%)	371678 (19%)
TME3	ACMV-CM	20	720629	30236 (4%)	23860 (3%)	6376 (1%)
TMS97/2205	ACMV-CM	20	743895	54973 (8%)	42707 (6%)	12265 (2%)
60444	ACMV-CM	20	829345	64084 (7%)	54566 (6%)	9518 (1%)
60444	ACMV-CM	45	574227	41466 (7%)	35328 (6%)	6138 (1%)
TME3	Non infected	–	1026983	158 (0.02%)	78 (0.01%)	79 (0.01%)
TME204	Non infected	–	787231	142 (0.02%)	71 (0.01%)	70 (0.01%)
TMS30572	Non infected	–	1026986	178 (0.02%)	95 (0.01%)	83 (0.01%)
