# Supplementary material for: Differential response of cassava genotypes to infection by cassava mosaic geminiviruses
Source: Virus Res. 2017 Jan 2;227:69–81. doi: 10.1016/j.virusres.2016.09.022 (PMC5130204; doi:10.1016/j.virusres.2016.09.022)
Supplement: Supplementary file 1 [file mmc1.docx]

| **Primer Name** | **Sequence** | **Size of the amplicon (bp)** | **Purpose** |
| --- | --- | --- | --- |
| ACMV-AC1-1538F | 5’CGGATGGCTCGCTTCTTGAATTGTC3’ | 938 | ACMV Detection |
| ACMV-AC1-2476R | 5’TCTGTAGGGAGCTGCATCAGAATGG3’ |  |  |
| ACMV-BC1-1354F | 5’ TCAGGTAGCTGGTGTAGCCCT 3’ | 805 | Detection |
| ACMV-BC1-2159R | 5’ GGTGTTGTGGCTTTGACACG 3’ |  |  |
| SP6-AMCV-AC1 | 5’ATTTAGGTGACACTATAGTGCTTACGCCAAAGCGCTTAACAG 3’ | 797 | RNA probe |
| AMCV-AC2 | 5’TACCCGCCATTCATTGCTTGAGGA 3’ |  |  |
| ACMV-CM AC1-probe-F | 5’ CCTGGATTGCAGAGGAAGATAG 3’ | 329 | DNA Probe |
| ACMV-CM AC1-probe-R | 5’ GACGAATGGGTCGCTGATAA 3’ |  |  |
| ACMV-CM BC1-probe-F | 5’ ATTGAGCACCAGGCGATATAC 3’ | 218 | DNA Probe |
| ACMV-CM BC1-probe-R | 5’ CACATAGAGGCAGTAGCCATAAA 3’ |  |  |
| EACMV K201 AC1-1608F | 5’ TGATGGGCACTTGAGAACAATGGC 3’ | 1048 | Detection |
| EACMV K201 AC1-2656R | 5’ ACGACAGCAAATATGCCAAGAGCC 3’ |  |  |
| EACMV K201 MP1-1260F | 5’ TGGCCTTGGTGCTTCGGATT 3’ | 747 | Detection |
| EACMV K201 MP1-2007R | 5’ ACCGCAACCAGGTCCCATTT 3’ |  |  |
| EACMV-K201 AC2F | 5’ CGTGGTGGGTGATTGCGAAATAGA 3’ | 734 | RNA probe |
| SP6-EACMV-K201 AC1 R | 5’ATTTAGGTGACACTATAGAACGCAGATCGAATCTTCCAGGCT 3’ |  |  |
| EACMV-K201 AC2 Probe-F | 5’ CTTGTCGTGGTGGGTGATT 3’ | 335 | DNA Probe |
| EACMV-K201 AC2 Probe-R | 5’ AATCCGGGACCAACATCATC 3’ |  |  |
| EACMV-K201 BC1 Probe-F | 5’ GGTCTAACTCTGGTGTGTGTAAT 3’ | 507 | DNA Probe |
| EACMV-K201 BC1 Probe-R | 5’ ACCTCCACTACTTCTCCTCTAC 3’ |  |  |
| EACMV K201 390 | 5’ GGTCTTCCCTGTACGACTATC 3 | 110 | qRT-PCR |
| EACMV K201 500: | 5’ GGAACTTGAAGTCTGGGTTTCC 3’ |  |  |
| EACMV K201 524 | 5’ GATGCGAATTTCACGCCTTC 3’ | 109 | qRT-PCR |
| EACMV K201 633 | 5’ ACCTCCACTACTTCTCCTCTAC 3’ |  |  |
| COX F | 5’ CGTCGCATTCCAGATTATCCA 3’ | 75 | qRT-PCR |
| COX R | 5’ CAACTACGGATATATAAGRRCCRRAACTG 3’ |  |  |
| DCL2-F3 | 5’ ATGCACACTGACCTCGTC 3’ | 110 | DCL2 qRT-PCR |
| DCL2-R3 | 5’ GTCATCACAAGCACCTCA 3’ |  |  |
| DCL3-F1 | 5’ CCTGGTGACTTGACGGATTT 3’ | 105 | DCL3 qRT-PCR |
| DCL3-R1 | 5’ CTTGTATAGCTCCCATCTCAC 3’ |  |  |
| DCL4-F3 | 5’ TGCTACTAAAGTGGGTGAAGAAG 3 | 109 | DCL4 qRT-PCR |
| DCL4-R3 | 5’ CGCACGTCCTCTAGATTGTATG 3’ |  |  |
| UBQ10 F | TGCATCTCGTTCTCCGATTG | 115 | Ubiquitin qRT-PCR |
| UBQ10 R | GCGAAGATCAGTCGTTGTTGG |  |  |

**Supplementary Table 1** List of forward and reverse primers employed in this study.
