# Supplementary material for: Differential response of cassava genotypes to infection by cassava mosaic geminiviruses
Source: Virus Res. 2017 Jan 2;227:69–81. doi: 10.1016/j.virusres.2016.09.022 (PMC5130204; doi:10.1016/j.virusres.2016.09.022)
Supplement: Supplementary file 2 [file mmc2.docx]

| Genomic region | EACMV KE2 (K201) | | ACMV CM | |
| --- | --- | --- | --- | --- |
|  | **Nucleotide position** | **%GC** | **Nucleotide position** | **%GC** |
| **Full genome A** | 1-2801 | 45.94 | 1-2777 | 44.98 |
| AV2 | 174-539 | 49.72 | 142-483 | 48.83 |
| AV1 | 334-1107 | 44.83 | 302-1075 | 44.83 |
| AC3 | 1104-1508 | 42.96 | 1072-1476 | 40 |
| AC2 | 1249-1656 | 50.98 | 1217-1624 | 46.07 |
| AC1 | 1565-2644 | 46.16 | 1533-2609 | 46.33 |
| AC4 | 2254-2487 | 48.71 | 2153-2575 | 46.80 |
| IR left | 2645-2801 | 43.31 | 1-141 | 42.78 |
| IR right | 1-173 | 55.49 | 2576-2777 | 51.06 |
| **Full genome B** | 1-2753 | 43.91 | 1-2726 | 40.60 |
| BV1 | 325-1125 | 45.06 | 443-1213 | 41.37 |
| BC1 | 1256-2275 | 46.56 | 1222-2118 | 43.58 |
| IR left | 1-181 | 49.44 | 2401-2726 | 35.88 |
| IR right | 2581-2753 | 44.50 | 1-241 | 51.66 |

**Supplementary Table 2** Percentage GC content of EACMV KE2 (K201) and ACMV CM genomic units.
