# Supplementary material for: Differential response of cassava genotypes to infection by cassava mosaic geminiviruses
Source: Virus Res. 2017 Jan 2;227:69–81. doi: 10.1016/j.virusres.2016.09.022 (PMC5130204; doi:10.1016/j.virusres.2016.09.022)
Supplement: Supplementary file 3 [file mmc3.pptx]

## Slide 1
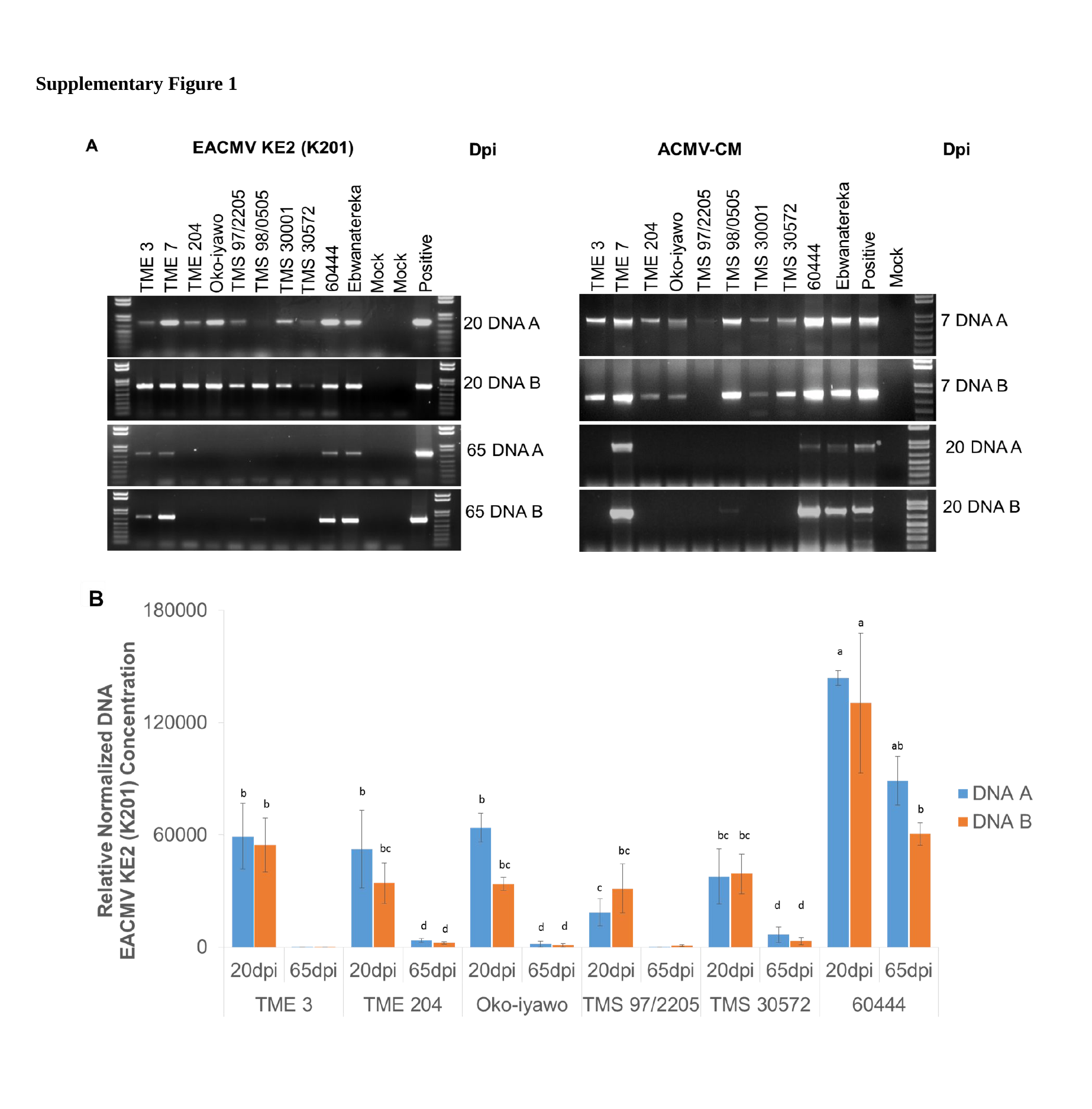

Supplementary Figure 1

## Slide 2
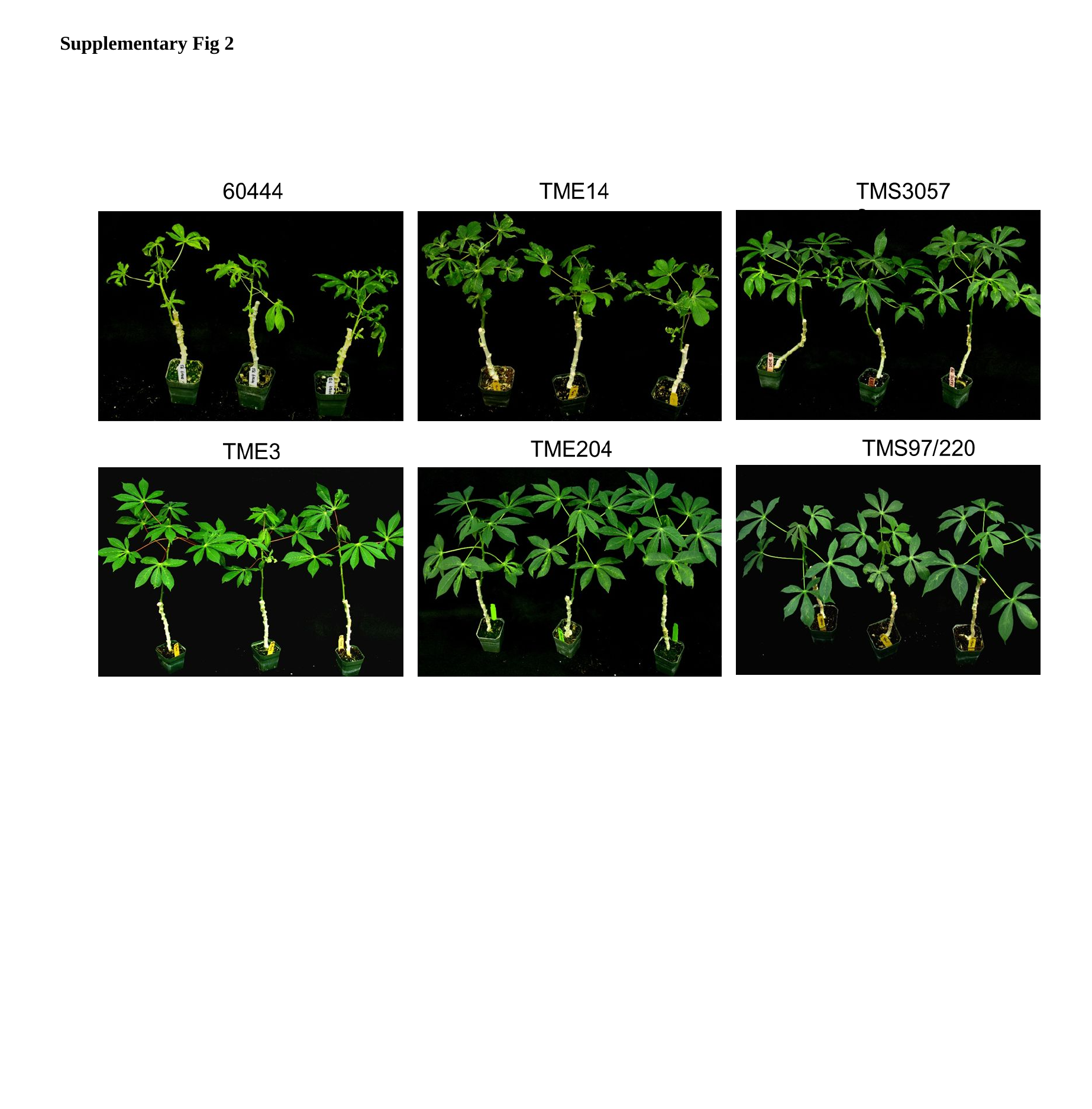

Supplementary Fig 2

## Slide 3
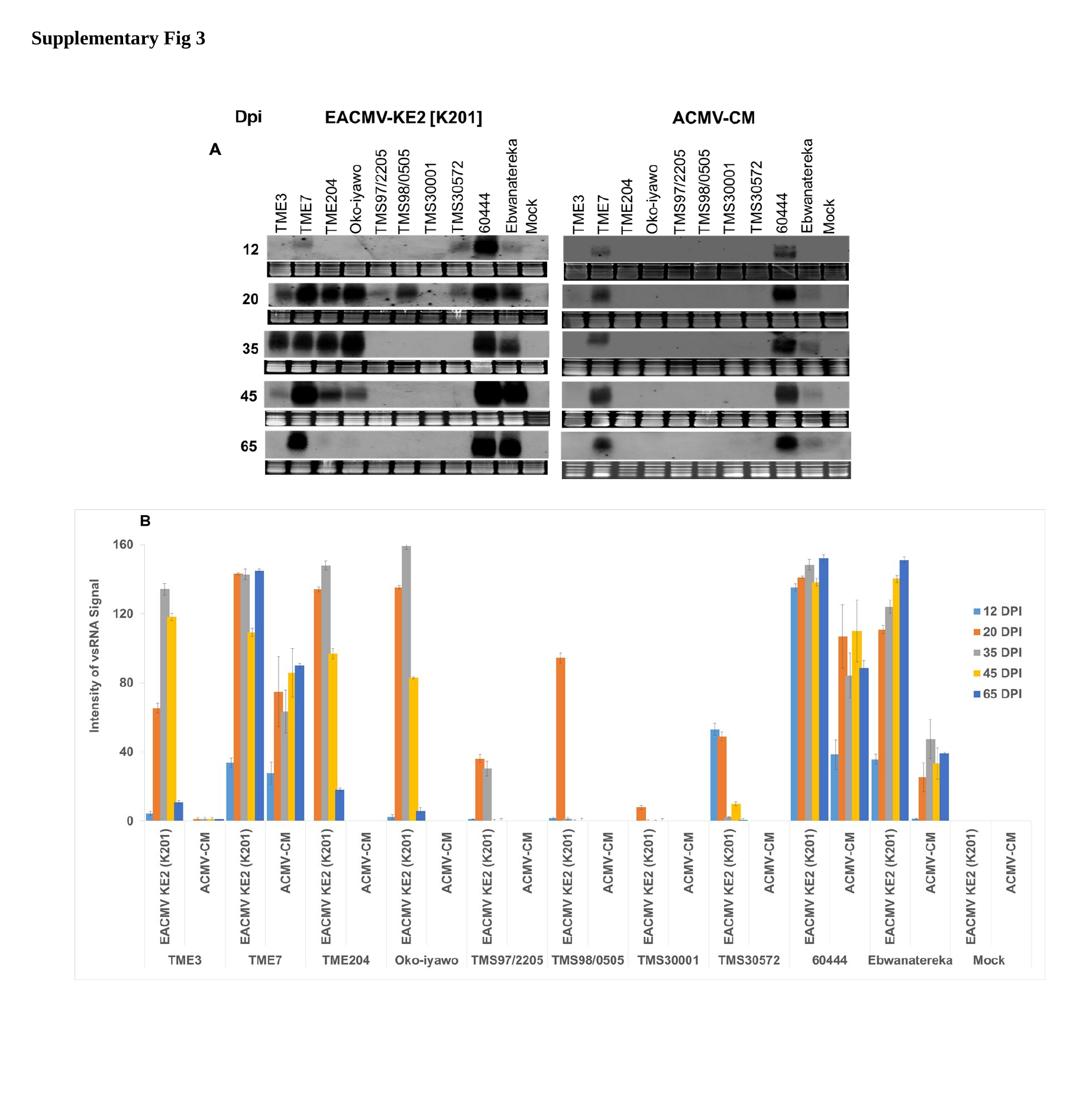

Supplementary Fig 3

## Slide 4
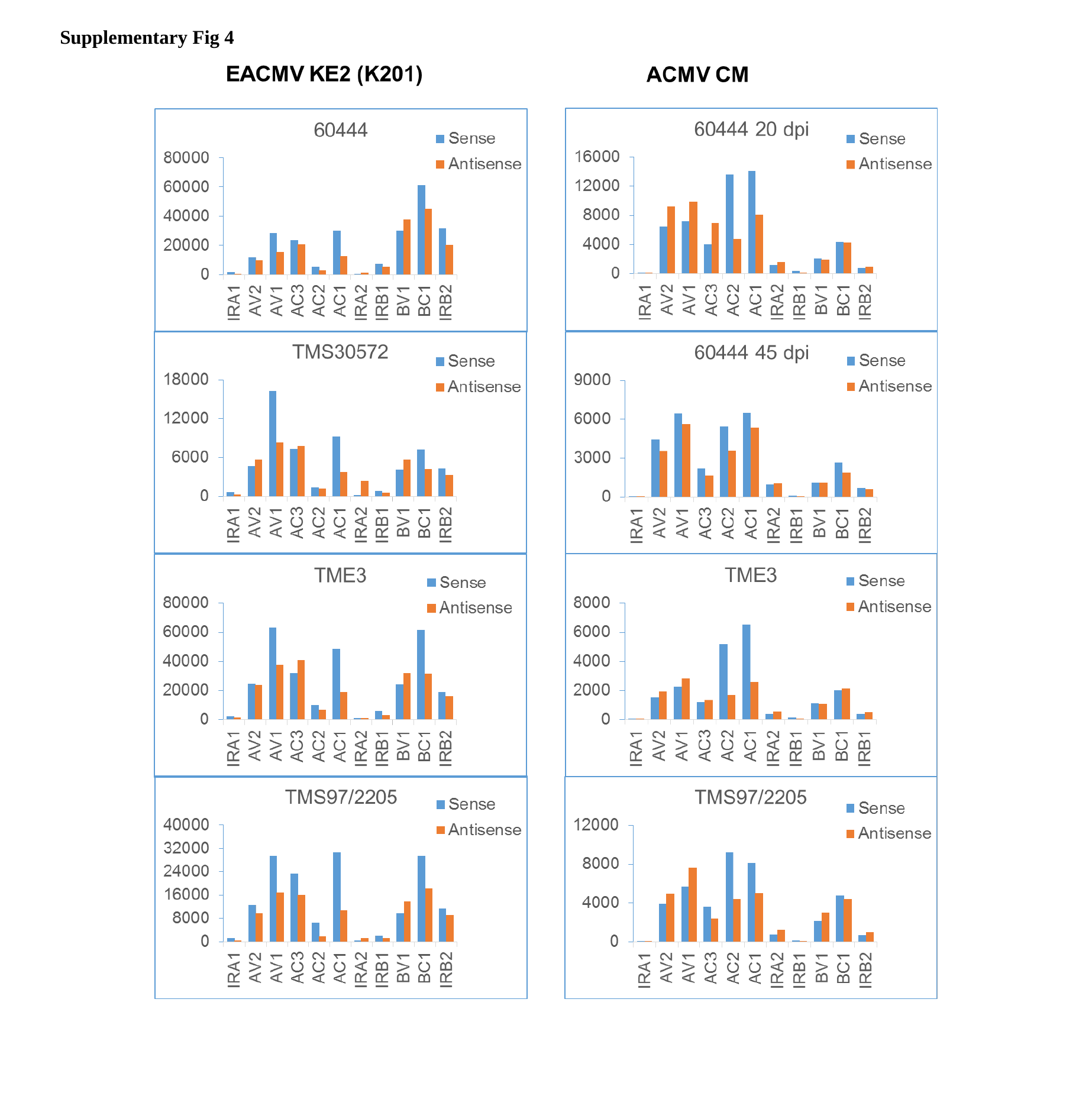

Supplementary Fig 4
